# Preparation and Catalytic Activity for Aerobic Glucose Oxidation of Crown Jewel Structured Pt/Au Bimetallic Nanoclusters

**DOI:** 10.1038/srep30752

**Published:** 2016-08-01

**Authors:** Haijun Zhang, Liqiong Wang, Lilin Lu, Naoki Toshima

**Affiliations:** 1 The State Key Laboratory of Refractories and Metallurgy, Wuhan University of Science and Technology, Wuhan 430081, China; 2 Advanced Materials Institute, Tokyo University of Science Yamaguchi, SanyoOnoda, Yamaguchi 756-0884, Japan

## Abstract

Understanding of the “structure-activity” relations for catalysts at an atomic level has been regarded as one of the most important objectives in catalysis studies. Bimetallic nanoclusters (NCs) in its many types, such as core/shell, random alloy, cluster-in-cluster, bi-hemisphere, and crown jewel (one kind of atom locating at the top position of another kind of NC), attract significant attention owing to their excellent optical, electronic, and catalytic properties. PVP-protected crown jewel-structured Pt/Au (CJ-Pt/Au) bimetallic nanoclusters (BNCs) with Au atoms located at active top sites were synthesized via a replacement reaction using 1.4-nm Pt NCs as mother clusters even considering the fact that the replacement reaction between Pt and Au^3+^ ions is difficult to be occurred. The prepared CJ-Pt/Au colloidal catalysts characterized by UV-Vis, TEM, HR-TEM and HAADF-STEM-EELS showed a high catalytic activity for aerobic glucose oxidation, and the top Au atoms decorating the Pt NCs were about 15 times more active than the Au atoms of Au NCs with similar particle size.

Tailoring the size, shape, structure, crystallinity, and composition of bi- and tri-metallic nanoclusters (BNCs/TNCs) and further providing an effective way to tune their performance for catalysis have attracted a great deal of attention in recent years^1^,^2^,^3^,^4^,^5^,^6^,^7^,^8^,^9^,^10^,^11^,^12^,^13^,^14^,^15^. It is accepted that the shape/structure of the BNCs and TNCs are of great importance for modern catalysis research, and that unraveling the complex interaction between shape/structure of the NCs and reactants and tailoring the catalytic activity at the atomic level are key steps towards gaining fundamental insight in catalysis^16^,^17^,^18^,^19^,^20^,^21^,^22^,^23^,^24^,^25^,^26^.

It has become clear that the high density of active sites of atomic steps, edges, and kinks on the surface of high-index facets are especially important for catalysis process^27^,^28^,^29^. For example, the oxidation current density on hexoctahedral and concave cubic Pt-Ni alloy nanocrystals with respectively exposed {h k l} and {hk0} high-index facets was almost 2.5 and 2.7 times higher than that observed for Pt black, and 3.6 and 3.9 times that of Pt/C in the electrooxidation of methanol, respectively^30^. The calculated initial activities of concave PtNi_3_ were respectively 7.9 and 10.5-times higher than those observed using octahedral PtNi_3_ and Pt_3_Ni^31^. On the other hand, it is also reported that urchin-like Pt_3_Ni nanocrystals exhibited a higher electrocatalytic activity for the reduction of I_3_^−^ to I^−^ as well as enhanced power conversion efficiency^32^, and the catalytic efficiencies of the Pt-Ni nanodendrites were remarkably superior to those Pt-Ni octahedra and concave nanostructures in terms of reaction times and yields using the same quantities of platinum^33^.

Catalysis over Pt/Au BNCs is of special interest among noble metal nanoalloys. Liu *et al*. prepared bimetallic Au–Pt/TiO_2_ for conversion of glycerol to lactic acid in alkaline aqueous solutions, and the results indicated that the bimetallic catalysts are stable and recyclable under the reaction conditions; however, the monometallic Au and Pt catalysts showed dramatic decreases in their activities^34^. Prati and coworks examined the catalytic activities of Pt/Au on carbon supports for liquid-phase oxidation of glycerol and n-octanol. The synergistic catalytic effect between Au and Pt was proved to be considerable^35^. Moreover, Pt/Au BNCs, such as PtAu alloy catalyst^36^, two-dimensional (2D) layerlike Pt-decorated Au nanostructures^37^,^38^ and Au–Pt core–shell nanoparticles (Au–Pt NPs)^39^,^40^,^41^, have been explored extensively as potential candidates for direct methanol fuel cells. Our group’s previous results also showed that the PVP-protected Pt/Au BNCs of about 1.5 nm in diameter exhibited nearly 10 times higher catalytic activities for aerobic glucose oxidation than that of Au nanoparticles (NPs) with nearly the same particle size^42^,^43^.

It is well-accepted that low-coordination atoms in the surface of NCs play a key role in defining the catalysis process^44^,^45^. In our previously published literatures, we already reported the preparation, characterization and catalytic activity of “crown jewel”-structured Pd/Au (CJ-Pd/Au) BNCs^46^,^47^ and (IrPt)/Au trimetallic NCs (CJ-(IrPd)/Au TNCs) via the galvanic replacement reaction between Pd (or mainly Pd) and Au^3+^ ions^48^, where the Au atoms were controllably reduced at the top site on the surface of Pd and IrPd mother clusters, and then exhibited a high catalytic activity towards aerobic glucose oxidation. The catalytic activity of the CJ-catalysts is completely site-specific, only the top atoms are effective for the catalysis, and the activities of top Au atoms tend to decrease with the increasing number of the neighboring coordinated Au atoms. Thus, the CJ-structure is the utmost limit of the morphological anisotropy and the top Au atoms are the utmost limit of the anisotropy in catalytic function.

Based on the results of our group and published literatures^42^,^43^,^46^,^47^,^48^, it is reasonably believed that Pt/Au BNCs with a CJ-structure should be also a highly active catalyst for aerobic glucose oxidation. However, as we know that the standard reduction potentials (*E*°s) in aqueous acidic solution at 25 °C of Au^3+^, Pt^4+^, and Pd^2+^ are +1.5, +1.2, and +0.987 V, respectively. This means that the galvanic replacement reaction between Pt and Au^3+^ should be more difficult than that between Pd and Au^3+^ in previously published literatures^46^,^47^,^48^. Thus, it is of great interest and challenge to prepare CJ-Pt/Au BNCs with a small particle size and a high activity. To the best of our knowledge, there is a no/few report on the galvanic replacement of Pt metal with Au^3+^ ion thanks to the small difference in redox potentials compared with that between Pd metal and Au^3+^ ion.

With aforementioned motivations, we examined the preparation of CJ-Pt/Au BNCs via galvanic replacement reaction from Pt mother NCs with a small average particle size in present paper. At first, Pt mother NCs with diameter of 1.4 nm were prepared by using rapid injection of NaBH_4_ method, and then converted to CJ-Pt/Au BNCs via a replacement reaction. Finally the characterization and catalytic activities towards aerobic glucose oxidation of the prepared CJ-Pt/Au BNCs were investigated. The catalytic activities for the glucose oxidation of CJ-Pt/Au, CJ-Pd/Au and CJ-(IrPd)/Au BNCs as well as Au NCs were compared with each other, and the possible reasons for the activity difference of these catalysts composed of different elements were discussed from a viewpoint of electronic structure.

## Experimental section

### Materials

Hydrogen tetrachloroaurate (III) tetrahydrate (HAuCl_4_·4H_2_O, 99.9%) purchased from Tokyo Kasei Kogyo, Ltd., hexachloroplatinic (IV) acid (H_2_PtCl_6_, 99.99%) and PVP (poly(*N*-vinyl-2-pyrrolidone, K35, molecular weight about 40,000) purchased from Wako Pure Chemical Industries, Ltd., were directly used without purification.

### Preparation of Pt “mother clusters” (PVP-protected Pt NCs)

It is well known that the replacement reaction between Pt and Au^3+^ ions is more difficult than that between Pd and Au^3+^, and that the smaller the metallic particle size is, the higher the reaction activity is. Thus, preparation of Pt mother clusters with size less than 1.8 nm is necessary for the synthesis of CJ-Pt/Au BNCs since the replacement reaction between Pd and Au^3+^ ions can be easily occurred for Pd mother clusters with size of 1.8 nm based on our previous results^46^,^47^. It is reported that Pt nanoparticles with size of 3.3 ± 1.6 nm, 2.7 ± 0.8 nm, and 1.4 ± 0.4 nm can be respectively prepared by using alcohol reduction method, dropwise addition of NaBH_4_ and rapid injection of NaBH_4_ method^43^. Hence, the dispersions of the PVP-protected Pt mother NCs with enough small size were synthesized by the method of rapid injection of NaBH_4_^43^. Typically, an aqueous solution of H_2_PtCl_6_ (50 mL, 1.32 mM) was added into an aqueous PVP solution (50 mL, 132 mM in monomer unit; The molar ratio of PVP in monomer units to the total metal ions is usually kept as 100, designed as R_PVP_ = 100.) and stirred in an ice-water bath at 0 °C for 15 min. Then, an aqueous solution of NaBH_4_ (20 mL, 16.5 mM, 0 °C) was rapidly injected into the above-mentioned solutions under vigorous stirring. The addition time of rapid injection of NaBH_4_ into PtCl_6_^2−^/PVP was within 5 s, and the mixture was stirred for another 1 h to obtain the colloidal dispersions of Pt mother clusters. After the reduction process is completely finished, 1 M-HCl solution was dropwise added into the prepared Pt dispersions and followed by kept stirring for 30 min to decompose the residual NaBH_4_. Although it is known that the Pt NCs can decompose BH_4_^−^ rapidly, however, the possibility of the catalytic decomposition could be low thanks to the short reaction time of present rapid injection of NaBH_4_ method.

The HRTEM, EDS mapping, and TEM image and size distribution histogram of the PVP-protected Pt NCs are shown in Fig. S1. The HRTEM images (Fig. S1a) demonstrate the prepared particles possess crystalline structures, and the EDS mapping (Fig. S1b) indicates the formation of Pt monometallic NCs. The average diameter of 1.4 nm of the prepared Pt NCs indicates that the NCs consist of about 55 atoms in a particle on average (Fig. S1c), and the mean particle diameter was used for the approximate calculation and preparation of the CJ-Pt/Au catalysts with a crown-jewel structure in the following steps.

### Preparation of Pt/Au catalysts with a “Crown-Jewel” structure

**Figure 1:**
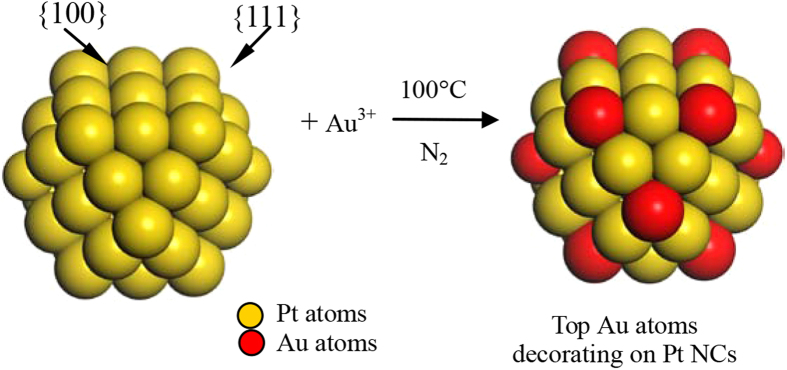
Schematic illustration of preparation of top Au atoms decorating Pt NCs by using a replacement reaction method.

Synthesis of the dispersions of CJ-Pt/Au NCs was carried out by a replacement reaction method^46^,^47^,^48^. Schematic illustration of the preparation process was shown in Fig. 1, and the detail compositions and preparation conditions are shown in Table S1. Typically, the CJ-1 NCs (the atomic ratio of Pt_55_ to the Au^3+^ in the synthetic solution is 55/3) were prepared as follows: An aqueous solution of HAuCl_4_·4H_2_O (20 mL, 0.180 mM) were dropwise added to an freshly-prepared Pt_55_ colloidal dispersion (50 mL, 1.32 mM) with continuous stirring at 100 °C and followed by heating for 30 min in an N_2_ atmosphere. The colloidal dispersions were washed using an ultrafilter membrane with a cutoff molecular-weight of 10,000 (Toyo Roshi Kaisha, Ltd.) twice with water and then once with ethanol under nitrogen to remove any extra reagents and by products. After remove of residual ethanol in the colloidal dispersions by using a rotary evaporator at 40 °C, powdery PVP-protected Pt/Au BNCs were finally obtained via vacuum drying at 40 °C for 48 h.

### Characterization of NCs

The UV–Vis (ultraviolet and visible light) absorption spectra were measured over the range of 200–800 nm by a Shimadzu UV-2500PC spectrophotometer.

TEM (Transmission electron microscopy) images were obtained using a JEOL TEM 1230 microscope at the accelerated voltage of 80 kV. For each sample, generally at least 200 particles from different parts of the grid were used to estimate the mean diameter and size distribution of the particles. High resolution transmission electron microscope (HRTEM) images were obtained using a JEM-2100UHR-STEM microscope (JEOL, Japan, 200 kV), and Energy dispersion X-ray spectroscopy (EDS) measurements was carried out with a NORAN UTW type Si(Li) semiconducting detector attached to the HRTEM equipment. The high-angle annular dark-field scanning TEM (HAADF-STEM) images were observed using a JEOL TEM 2010F microscope equipped with CEOS spherical aberration correctors at the accelerating voltage of 120 kV in the UBE Scientific Analysis Laboratory (Japan). High-resolution electron energy loss spectroscopy (EELS) measurements were carried out using an ENFINA1000 (Gatan, Inc.) detector with a beam diameter of about 0.22 nm attached to the HAADF-STEM equipment.

The metal content of the PVP-protected CJ-Pt/Au NCs was determined by ICP-OES (optical emission spectroscopy with inductive coupled plasma, Varian 720-ES).

XPS measurement was carried out by a Quantum 2000 spectrometer (PHILIPS) using the Al Kα radiation *(E* = 1486.6 eV). The binding energies were calibrated using the adventitious carbon contamination C1s feature at 284.6 eV as a standard. The existence of Au and Pt was monitored using the binding energy of Au 4f_7/2_ and Pt 4f_7/2_ features. The XPS characterization of Au and Pt was difficult for present CJ-Pt/Au BNCs since a lot of PVP (R_PVP_ = 100.) was used as protective reagent to assure the formation of CJ-Pt/Au BNCs with size less than 2 nm by present replacement reaction method, which make the contents of Au and Pt in the BNCs for XPS test is very low (Au: about 0.4 atom%; Pt: about 1.3 atom%). Decreasing the amount of PVP can certainly make the XPS characterization easy, but it will increase the size of the prepared BNCs and subsequently change its electronic structure.

### Catalytic properties for glucose oxidation at controlled pH

The catalytic performance of all the catalysts was evaluated using the glucose oxidation as the model reaction. The reactions were carried out at 60 °C in a 50-mL glass beaker placed in a thermostat (about 2000 mL). During the experiment, the pH of the reaction suspension was kept constant at 9.5 by the addition of a 1 mol L^−1^ NaOH solution using an automatic potentiometric titrator (Kyoto Electronics MFg., Co., Ltd., Japan). Oxygen was bubbled through the suspension at the flow rate of 100 mL min^−1^. The suspension was vigorously stirred by a magnetic stirrer. The starting concentration and volume of the glucose solution was 0.264 mol L^−1^ and 30 mL, respectively, and the charged weight of the catalyst was about 2 mg. The catalytic reactions were automatically carried out for 2 h. The initial specific catalytic activity related to the metal content of the NCs was calculated from the slope of a straight line fitted using the NaOH amount vs. reaction time curve. A typical NaOH amount vs. time diagram with the corresponding fit line is shown in Fig. S2.

The catalytic activities of all the samples were measured at least twice under the same conditions, and the TOF values of the top Au atoms were approximately calculated by the following equation,


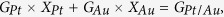


where *G*_*Pt*_ and *G*_*Pt/Au*_ are the evaluated catalytic activities of the Pt mother clusters and CJ-Pt/Au NCs, respectively. *X*_*Pt*_ and *X*_*Au*_ which were measured by ICP-OES are the atomic ratios of Pt and Au in the CJ-Pt/Au NCs, respectively. *G*_*Au*_ is the calculated catalytic activity of the Au atoms.

## Results and Discussion

**Figure 2:**
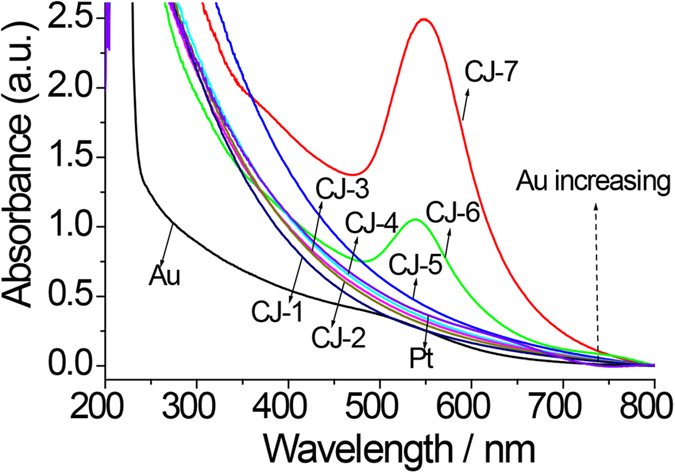
UV-Vis spectra of colloidal dispersions of Pt, Au, and CJ-Pt/Au NCs series catalysts.

Using 1.4-nm Pt NCs as mother clusters, a series of CJ-Pt/Au BNCs were prepared by galvanic replacement reaction method. The UV-Vis absorption spectra of the aqueous dispersions of a series of CJ-Pt/Au BNCs with various contents of Au accompanied with Pt_55_ mother clusters and Au NCs (prepared by rapid injection of NaBH_4_, the average size is about 1.4 nm as shown in ref. 43) are shown in Fig. 2. The absorbance of the Pt_55_ mother cluster dispersion monotonically increases with wavelength decreasing in the measured range from 800 to 200 nm. The small peak around 520 nm in the spectrum of the Au NCs dispersion is attributable to the surface plasmon resonance of the metallic Au. As for the prepared CJ-Pt/Au BNCs of CJ-1, CJ-2, CJ-3, CJ-4 and CJ-5, their absorbance increases with Au content increasing even though the geometrical shape of these spectra is still quite similar to that of Pt_55_ mother cluster dispersion. These changed absorptions can provide an indirect proof that the surface compositions of these CJ-Pt/Au BNCs are different from that of the Pt_55_ mother nanoclusters. Moreover, the absence of the surface plasmon peak of Au at about 520 nm for these NCs suggests that Au atoms prefer to deposit in several certain sites rather than all the surface of the Pt_55_ NCs. However, the clear plasmon peaks around 550 nm observed for the CJ-6 and CJ-7 NCs are suggestive of the formation of an enough amount of large Au nanoparticles or the coverage of the surface of Pt NCs with an enough amount of Au atoms in these two samples.

Mapping EDS attached to HRTEM was carried out to confirm the formation of the Pt/Au BNCs (Fig. S3). It indicated that the atomic ratios of Pt: Au for CJ-2 and CJ-3 samples were 96:3 and 39:13, respectively. These results provided direct proof that Pt/Au BNCs were indeed formed by using present galvanic replacement reaction method.

**Figure 3:**
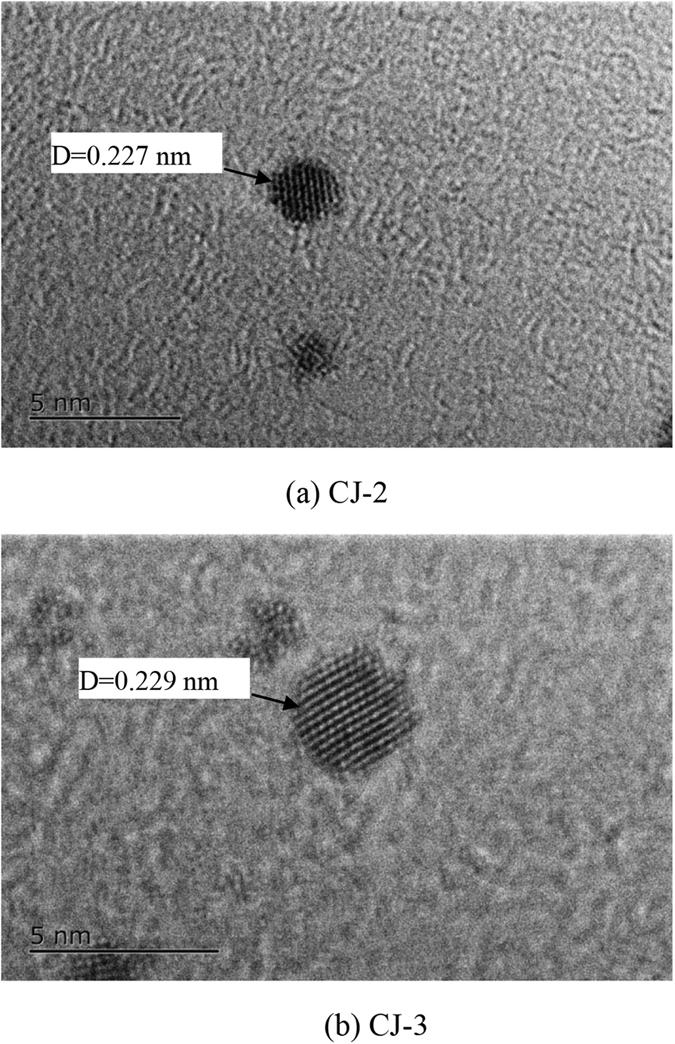
HRTEM micrograph of CJ-2 and CJ-3 BNCs.

Figures 3 and S4 shows HRTEM images of CJ-2 and CJ-3 samples. As revealed by the lattice fringes shown in Fig. 3, the particles possess crystalline structures. It also indicates that the measured interplanar distances of the individual random-chosen particles of CJ-2 and CJ-3 samples are 0.227 nm (Fig. 3a) and 0.229 nm (Fig. 3b), respectively. These measured values do not match with those interplanar distances of pure Au or Pt shown in Table S2. However, they lie between the interplanar spacing of Pt (111) (0.2265 nm) and that of Au (111) (0.2355 nm), suggesting the formation of Pt/Au bimetallic NCs (Table S3).

**Figure 4:**
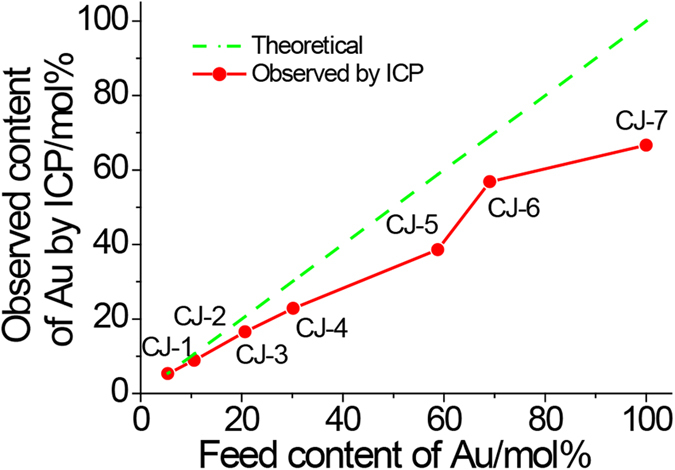
Composition data of Au content in CJ-Pt/Au NCs derived from ICP analysis. (The plot shows the relationship between the Au% from the synthetic feed and Au% in the final NCs).

**Figure 5:**
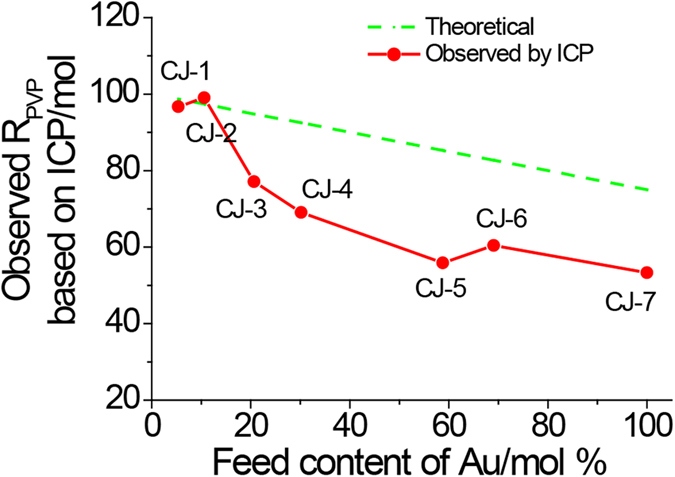
Comparison of PVP contents in CJ-Pt/Au NCs before and after replacement reaction. (The plot shows the relationship between the PVP from the fed solution and PVP in the final NCs. When the Au^3+^ content is high, the large difference between the theoretical and actual R_PVP_ shows that a lot of the PVP is consumed for reduction of the Pt and Au nanoparticles. R_PVP_: molar ratio of PVP in monomer units to all the metal ions in the samples).

Chemical composition measurement of the prepared CJ-Pt/Au NCs was carried out by using ICP-OES to get information of the real content of Au element in these samples. Figure 4 showed the metal compositions of the prepared CJ-Pt/Au NCs measured by ICP analysis which indicates that there is a slight difference between the Au contents in the final NCs and those in the synthetic feeding. Based on the results in Fig. 4 and Table S4, it can be concluded that: 1) Au was indeed reduced in the BNCs via present replacement reaction way; 2) Clear deviations between the final Au content and the feed can be observed for the as prepared BNCs. The higher the Au content, the larger the deviation. We think the larger differences between the final Au content and the feed for the samples of CJ-4, CJ-5, CJ-6 and CJ-7 can be attributed to that some of the Pt^4+^ ions formed by the replacement reaction were reduced by PVP and deposited again on the NCs. This suggestion can be further confirmed by the plot of the PVP content in the final CJ-Pt/Au BNCs which clearly indicates the decomposition of PVP during the replacement reduction process (Fig. 5).

**Figure 6:**
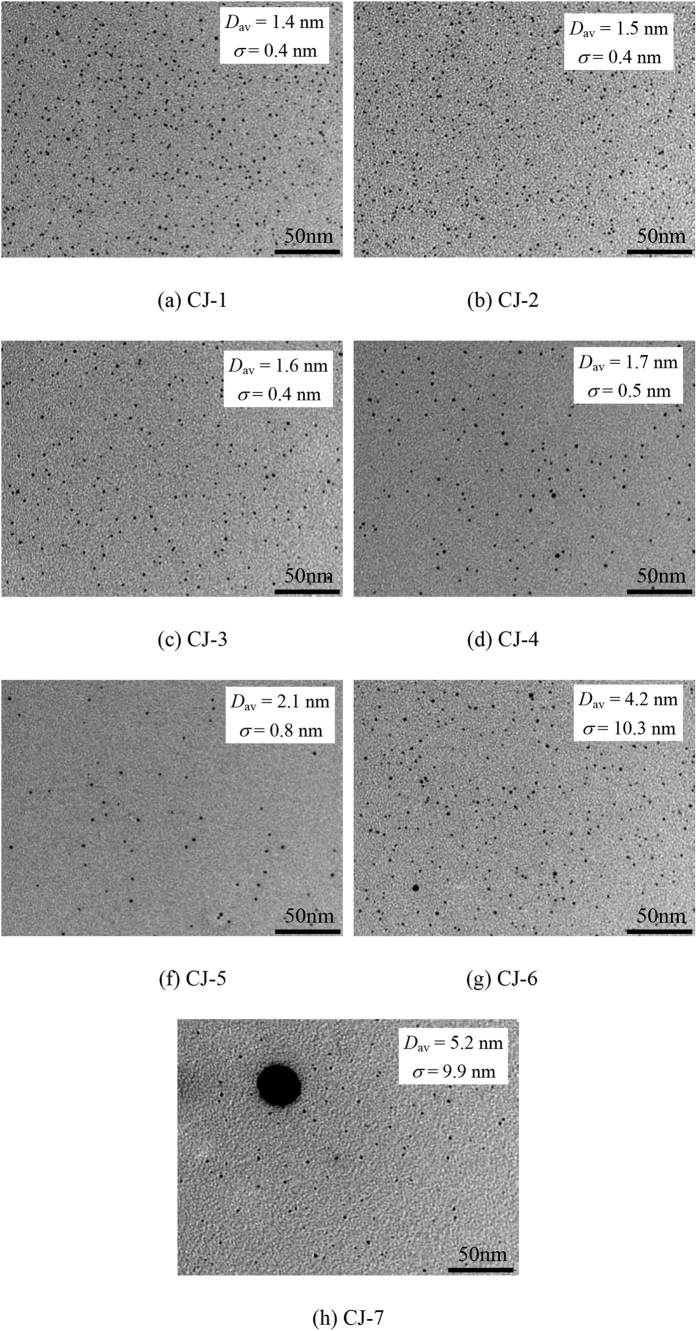
TEM micrograph of CJ-Pt/Au NCs series catalysts prepared by replacement reaction method.

Figure 6 showing typical TEM images of the CJ-Pt/Au BNCs (Size distribution histograms are shown in Fig. S5) reveals that, as for CJ-1, CJ-2, CJ-3 and CJ-4 NCs, all the clusters are spherical and well-isolated, and their average sizes (± standard deviation) are 1.4 ± 0.4 nm, 1.5 ± 0.4 nm, 1.6 ± 0.4 nm and 1.7 ± 0.5 nm, respectively. As for the samples of CJ-5, CJ-6 and CJ-7 NCs, especially in the case of the CJ-8 NCs, however, large particles with diameter more than 10 nm accompanied with small particles less than 2 nm were observed at the same time. The average particles sizes of CJ-5, CJ-6 and CJ-7 are 2.1 ± 0.8 nm, 4.2 ± 10.3 nm, and 5.2 ± 9.9 nm, respectively. The formation of large particles more than 10 nm during the replacement reaction process can be ascribed to the synergistic effect of Oswald ripening and the presence of a large amount of PVP in present experiments. It is well-known that the former can cause dissolution of the small particles and the growth of large ones^49^,^50^, and the latter can cause the reduction of the Pt^4+^ ions produced by the replacement and result in the *in-situ* deposition of the formed Pt again on the NCs, because the PVP can work as a weak reduction agent^51^. These results also provide an evidence for the deviation of the PVP content between the fed solution and the final samples shown in Fig. 5.

**Figure 7:**
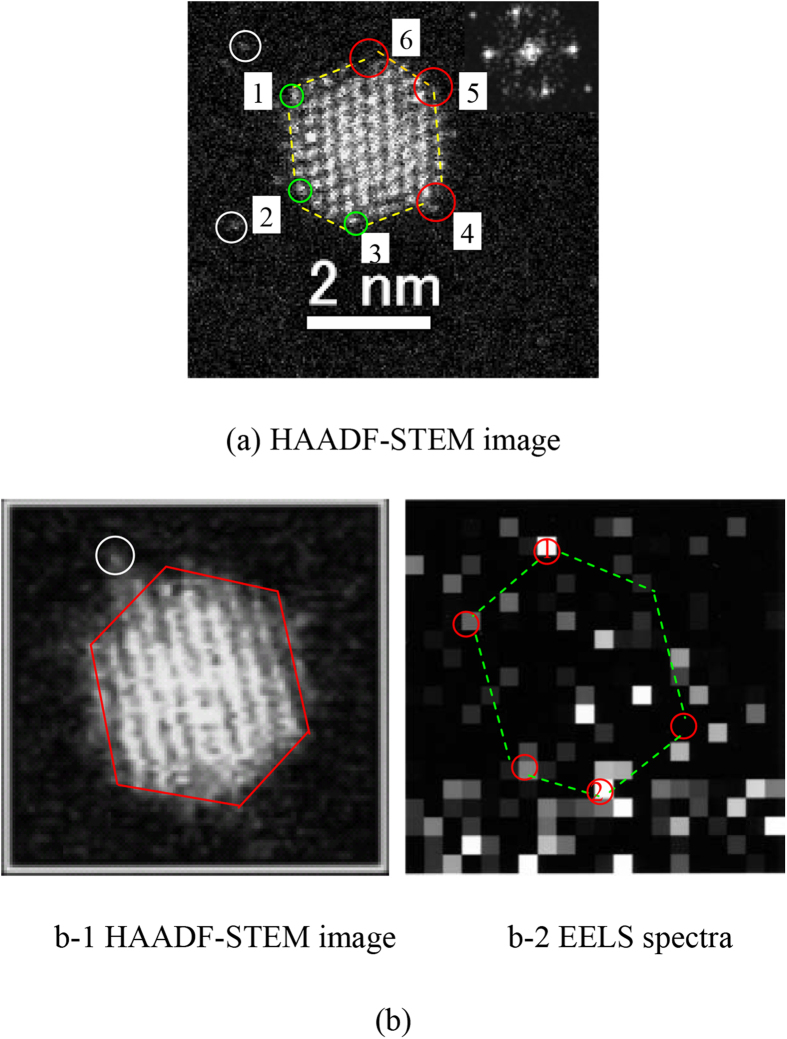
Microstructure characterization of CJ-Pt/Au NCs (CJ-1). (**a**) HAADF-STEM image of a single CJ-Pt/Au NCs (**b**) HAADF-STEM image and EELS mapping (4.32 × 4.32 nm, 20 × 20 pixels, 0.22 nm/pixel.). The dotted yellow hexagon in (**a**) draws the shape of the cluster, the green circles indicate the top atoms with orderly arrangement which should be the unreacted Pt atoms, the red circles show the presence of the vacancies arisen from the replacement reaction, and the white circle shows the moving single atom. The dotted green hexagon in (b-2) draws a shape of the cluster estimated based on EELS results, and the red circles marked with No. 1 and No. 2 in (b-2) probably indicates the presence of the top Au atoms.

HAADF-STEM and EELS observations were carried out to further study the structure and composition of the prepared CJ-Pt/Au BNCs. A HAADF-STEM image shown in Fig. 7a clearly demonstrates the column arrangements of Pt and Au atoms in a CJ-Pt/Au NCs with size of about 2 nm in diameter, and the insert fast Fourier-transform (FFT) pattern in Fig. 7a shows the cluster is a single crystal with its surface being enclosed by both {111} and {100} facets. Close investigation of the image further reveals that clear vacancies can be also observed in the NCs in corners 4-6, the presence of these vacancies (Figs 7a and S6) can give the reasonable evidences that the replacement reaction between Pt_55_ NCs and Au^3+^ ions occurs initially at the top sites of the mother clusters.

EELS mapping was carried out in detail to verify the *in-situ* reduced Au atoms located at the top site of the Pt_55_ mother cluster or not. Since the electron beam size of EELS (about 0.22 nm) is less than that Au atom diameter (0.268 nm), the Au atoms in the surface of the BNC can be sorted one by one, and the existence of Au atoms is expressed by the bright squares shown in Fig. 7b. Even though the Au EELS map (Fig. 7b-2) of the NC which HAADF-STEM image is shown in Fig. 7b-1, indicates that Au atoms are not distributed in orderly way in the NC and seem to move within a certain area, it can be still concluded by comparing the Au EELS map (Fig. 7b-2) with the shape of the characterized NC (Fig. 7b-1) that at least parts of the reduced Au atoms are located at the top site of the cluster. On the other hand, the distribution of Au atoms in a certain area can also provide an indirect proof of the presence of single Au atoms in the present CJ-Pt/Au BNCs, because Au atoms near the tops of the BNCs easily move under electron beam during the STEM observation^46^,^47^.

**Figure 8:**
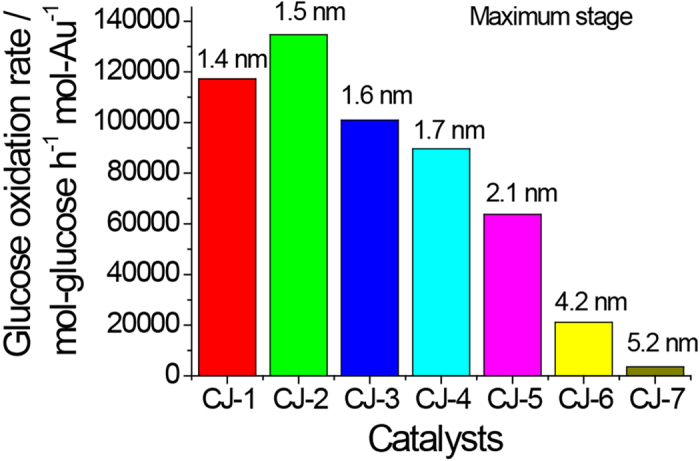
Catalytic activity of a series of CJ-Pt/Au BNCs for aerobic glucose oxidation. (Glucose/Au = 36500, mol ratio; the average particle size of each NC is indicated at the top of the corresponding bar).

**Figure 9:**
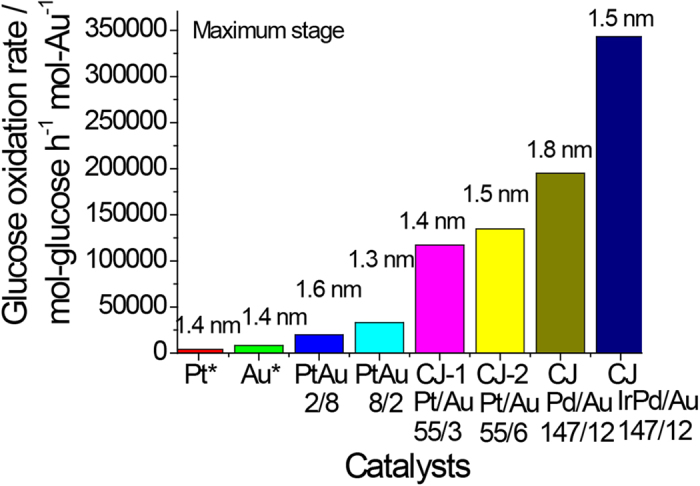
Comparison of the catalytic activity of CJ-Pt/Au, CJ-Pd/Au, CJ-IrPd/Au, Au, Pt, and Pt/Au alloy NCs for aerobic glucose oxidation. (Glucose/Au = 36500, mol ratio; Numbers shown at the top of each bar indicate the average particle sizes of the NCs. The activity of Au* and Pt* was normalized by the number of surface Au and Pt atoms, respectively).

Catalytic activity for aerobic glucose oxidation of the prepared CJ-Pt/Au BNCs was evaluated in water at 60 °C at a pH of 9.5. The initial catalytic activity of the CJ-Pt/Au BNCs shown in Fig. 8 indicates that the activities of the Au atoms decrease with Au content increasing, and that the maximum catalytic activity of the top Au atoms is about 134,700 mol-glucose·h^−1^·mol-Au^−1^ (CJ-2). Comparison of the activity of the prepared CJ-Pt/Au BNCs with that of Au, Pt, and PtAu alloy NCs shown in Fig. 9 indicates that the maximum catalytic activity of the top Au atoms of CJ-Pt/Au BNCs is about 15 times higher than that of the monometallic Au NCs (even if the activity was normalized to the surface atoms), and 4 to 32 times higher than that of the PtAu alloy BNCs (Pt_20_Au_80_ and Pt_80_Au_20_) and Pt monometallic NCs, respectively, although the average particle sizes of all these NCs are about 1.4 nm. (TEM images of the PtAu alloy NCs are not shown here). The much higher catalytic activity of the Au atoms in the prepared CJ-Pt/Au BNCs than that of Au monometallic NCs and the alloy-structured PtAu BNCs provides an indirect proof of the formation of the unique crown-jewel structure.

Electronic charge transfer effects between different kinds of neighboring elements were always regarded as the possible reasons for the high catalytic activities of BNCs^12^,^13^,^14^,^47^. Owing to the relatively low ionization energy of Pt (9.02 eV) compared with that of Au (9.22 eV), Pt atoms should theoretically donate electrons to its neighboring Au atoms via the electronic charge transfer effect, which would then render the Au atoms electron sufficient in the CJ-Pt/Au BNCs. To confirm the existence of the negatively charged top Au atoms and further investigate the relationship between the electronic structure and the activity of present CJ-Pt/Au BNCs, the XPS measurement was carried out. The spectrum shown in Fig. S7 indicates that the elements of C, O, N, Au, Pt and Si can be detected from CJ-3 BNCs. The presence of C, O, and N can be attributed to the presence of PVP, and Si is caused by the substrate for the XPS sample. Even though the peak of Au and Pt in the XPS spectrum is very weak as expected and seen from the results shown in Fig. S7, the electron apparent binding energy (BE) of Au 4f_7/2_ (83.16 eV) in the sample is lower than that of the bulk Au (84.0 eV). The negative shift in the Au 4f BE suggests that a negative charge is deposited on the Au atoms of the CJ-Pt/Au BNCs, and provides an evidence that Au atoms are indeed negatively charged. In the case of Pt atoms, XPS results show that it is positively charged. Since it is accepted that the negatively charged Au atoms, which can generates hydroperoxo-like species from O_2_, play a key role in the oxidation of the glucose, we think that the high catalytic activity for glucose oxidation of the present CJ-Pt/Au BNCs comparing with that of Au NCs can be ascribed to the existence of negatively charged Au atoms in the BNCs.

Comparing the catalytic activities of present CJ-Pt/Au BNCs with that previously published CJ-Pd/Au and CJ-(IrPd)/Au NCs can reveal that the top Au atoms of present CJ-Pt/Au BNCs show a much lower activity for glucose oxidation than that of the top Au atoms of CJ-Pd/Au (194,980 mol-glucose·h^−1^·mol-Au^−1^) and CJ-(IrPd)/Au (343,190 mol-glucose·h^−1^·mol-Au^−1^) NCs (Fig. 9), and the reasons for it are still unclear for us at present stage. We infer that the electron density of the top Au atoms caused by electronic charge transfer effects may play a very important role on the catalytic activity for aerobic glucose oxidation even though we have no strict evidence on it.

## Summary and Conclusion

“Crown-jewel” structured Pt/Au BNCs were prepared via replacement reaction method using Pt NCs with an average diameter of 1.4 nm as mother clusters even though it is well-known that the replacement reaction between Pt and Au^3+^ ions is difficult to occur. The results of HAADF-STEM and EELS map indicated that at least parts of the Au atoms are located at the top site of the cluster. The catalytic activity for the aerobic glucose oxidation of the prepared CJ-Pt/Au BNCs is as high as 134,700 mol-glucose·h^−1^·mol-Au^−1^, which is 15 times higher than that of the monometallic Au NCs, and more than 4 times higher than the PtAu alloy BNCs with nearly the same particle size. Even though the catalytic activity of the CJ-Pt/Au BNCs was not so high as the cases of CJ-Pd/Au BNCs and CJ-(IrPd)/Au TNCs, the CJ structure is still effective for the activity enhancement. The high activity of the prepared CJ-Pt/Au BNCs can be attributed to the existence of negatively charged top Au atoms. This fundamental understanding shows that morphological and electronic control of bimetallic nanoclusters is very important for the development of the next generation of highly efficient catalysts.

## Additional Information

**How to cite this article**: Zhang, H. *et al*. Preparation and Catalytic Activity for Aerobic Glucose Oxidation of Crown Jewel Structured Pt/Au Bimetallic Nanoclusters. *Sci. Rep.* **6**, 30752; doi: 10.1038/srep30752 (2016).

## Supplementary Material

Supplementary Information
