# Supplementary material for: Preparation and Catalytic Activity for Aerobic Glucose Oxidation of Crown Jewel Structured Pt/Au Bimetallic Nanoclusters
Source: Sci Rep. 2016 Aug 1;6:30752. doi: 10.1038/srep30752 (PMC4967863; doi:10.1038/srep30752)
Supplement: Supplementary Information [file srep30752-s1.doc]

**Supporting information**

**Preparation and Catalytic Activity for Aerobic Glucose Oxidation of Crown Jewel Structured Pt/Au Bimetallic Nanoclusters**

Haijun Zhang^1^*, Liqiong Wang^1^, Lilin Lu^1^, and Naoki Toshima^2*^

^1^The State Key Laboratory of Refractories and Metallurgy, Wuhan University of Science and Technology, Wuhan 430081, China

^2^Department of Applied Chemistry, Tokyo University of Science Yamaguchi, SanyoOnoda, Yamaguchi 756-0884, Japan

*Corresponding author;

E-mail of the corresponding author:

Prof. Haijun Zhang: [zhanghaijun@wust.edu.cn](mailto:zhanghaijun@wust.edu.cn);

Prof. Naoki Toshima: toshima@rs.tus.ac.jp;

***Table S1* Preparation conditions of CJ-Pt/Au NCs by replacement reaction method**

| Code | Preparation conditions of Au atom | |
| --- | --- | --- |
|  | Pt/Au^3+^, atom ratio | Conditions |
| CJ-1 | 55/3 | 100°C/30 min |
| CJ -2 | 55/6 | 100°C/30 min |
| CJ -3 | 55/12 | 100°C/30 min |
| CJ -4 | 55/18 | 100°C/30 min |
| CJ -5 | 55/38 | 100°C/30 min |
| CJ -6 | 55/46 | 100°C/30 min |
| CJ -7 | 55/73 | 100°C/30 min |

3Pt + 4Au^3+^ = 4Au + 3Pt^4+^

***Table S2*** Theoretical lattice spacing of bulk Au and Pt with various reflection planes.

| Reflection plane | Lattice spacing, nm | |
| --- | --- | --- |
|  | Au | Pt |
| (111) | 0.2355 | 0.2265 |
| (200) | 0.2040 | 0.1962 |
| (220) | 0.1442 | 0.1387 |
| (311) | 0.1230 | 0.1183 |
| (222) | 0.1178 | 0.1133 |

(Au: JCPDS 89-3697; Pt: JCPDS 04-0802)

***Table S3*** Lattice spacing and indexed reflection planes of CJ-2 and CJ-3 BNCs determined by HR-TEM

| Samples | Measured Lattice  spacing, nm | Remarks | Indexed reflection plane of the BNCs |
| --- | --- | --- | --- |
| CJ-2 | 0.227 | In the range of Au(111) and Pt(111) | (111) |
| CJ-3 | 0.229 | In the range of Au(111) and Pt(111) | (111) |

***Table S4* Comparison of theoretical and measured Au contents of the prepared CJ-Pt/Au NCs.**

| Code | Theoretical contents | | ICP contents, mol |
| --- | --- | --- | --- |
|  | Starting  Pt/Au^3+^, mol | Theoretical Au content, wt% | Final  Au%, wt% |
| CJ-1 | 55/3 | 5.37 | 5.31 |
| CJ -2 | 55/6 | 10.60 | 8.87 |
| CJ -3 | 55/12 | 20.65 | 16.53 |
| CJ -4 | 55/18 | 30.19 | 22.83 |
| CJ -5 | 55/38 | 58.79 | 38.61 |
| CJ -6 | 55/46 | 69.05 | 56.82 |
| CJ -7 | 55/73 | 100 | 66.67 |

3Pt + 4Au^3+^ = 4Au + 3Pt^4+^


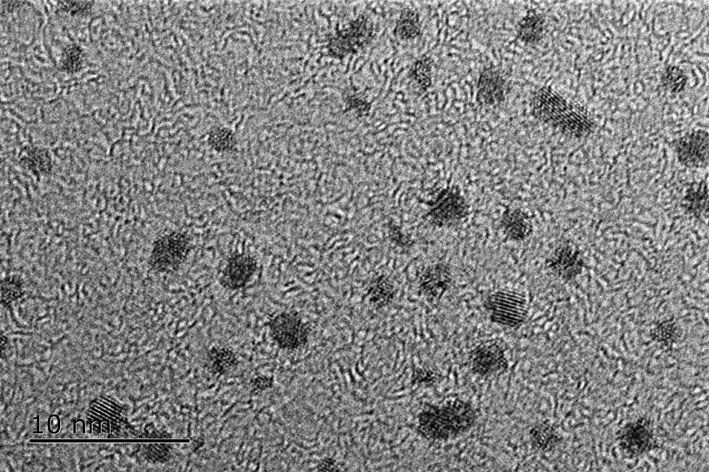

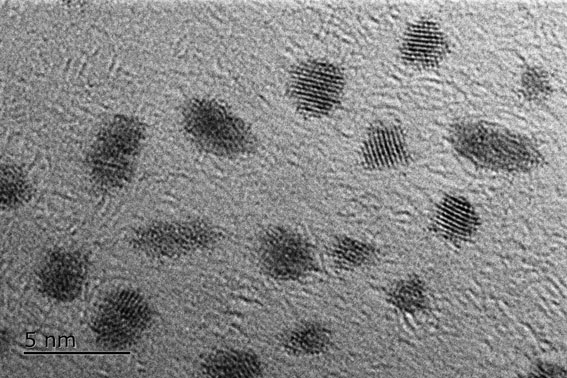


D=0.225 nm

(a) HRTEM


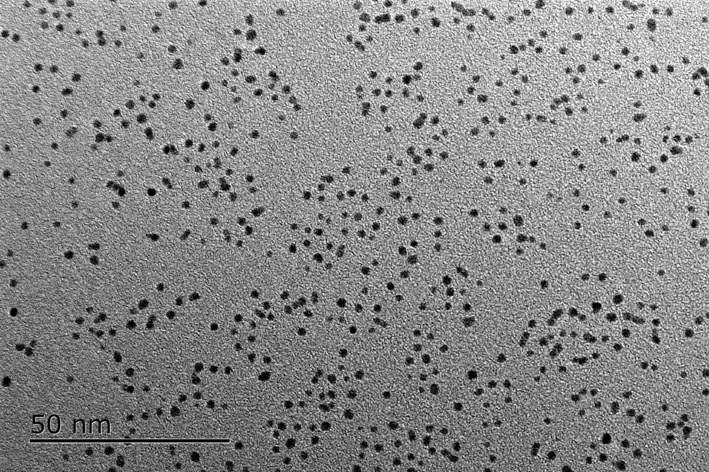

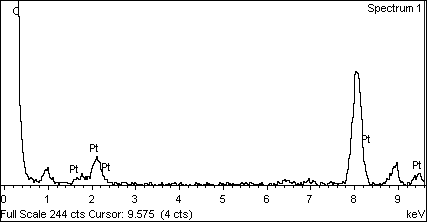


(b) TEM-mapping EDS


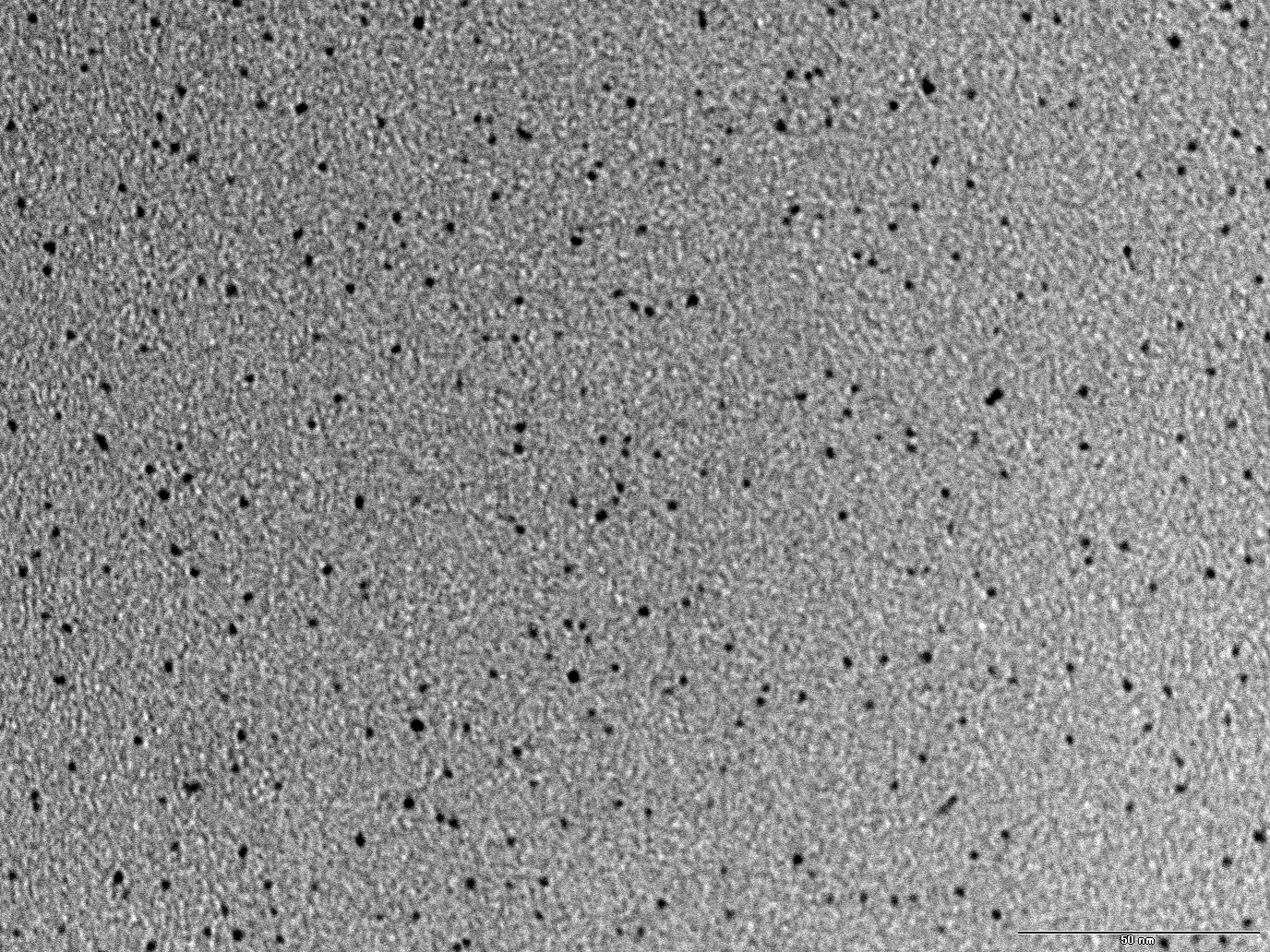


50nm

(c) TEM micrograph and size distribution

***Figure S1.*** HRTEM (a), mapping EDS (b), and TEM micrograph and size distribution (c) of Pt mother clusters prepared by rapid injection of NaBH_4_ (1.32mM, *R*_PVP_=100).

***Figure S2*.** The NaOH amount -time curve for glucose oxidation over “top Au atoms supported on Pt clusters” catalyst prepared by replacement reaction (CJ-2) and a fit straight line for determining TOF (Glucose/Au=36500, mol ratio; O_2_: 100ml/min; about 0.0020g catalyst was used for evaluation.).


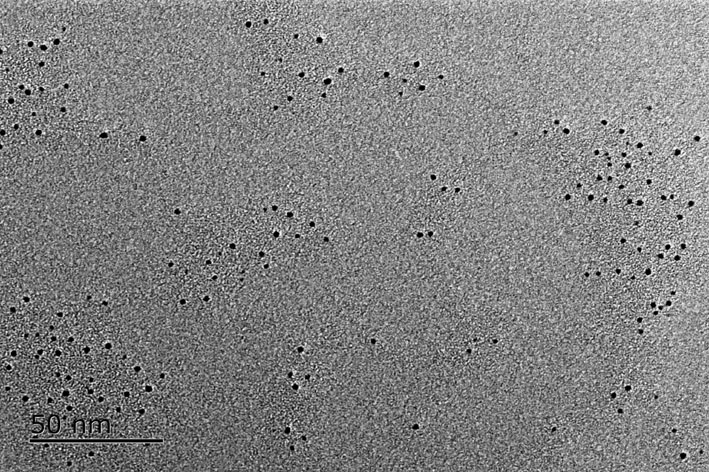


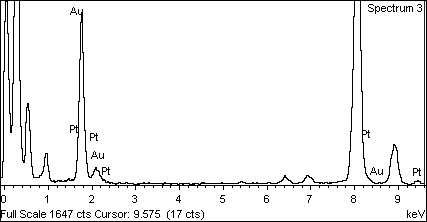

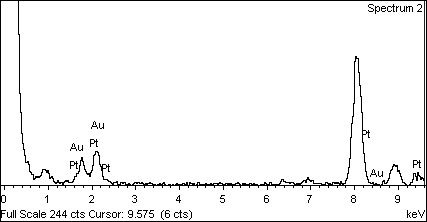


(a) CJ-2


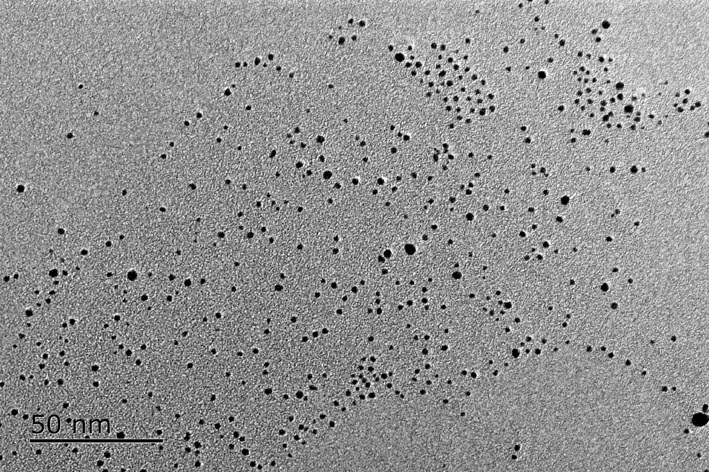


(b) CJ-3

***Figure S3***. TEM and mapping EDS of CJ-2 and CJ-3 BNCs


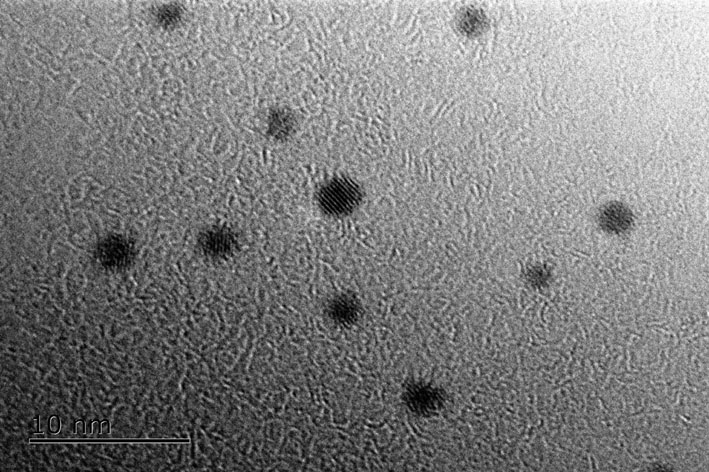

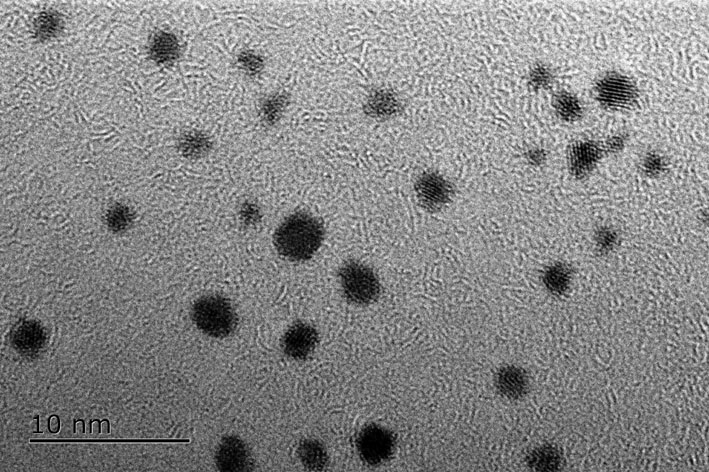


(a) CJ-2 (b) CJ-3

***Figure S4.*** HRTEM micrograph of CJ-2 and CJ-3 BNCs

(a) CJ-1 (b) CJ-2

(c) CJ-3 (d) CJ-4

(f) CJ-5 (g) CJ-6

(h) CJ-7

***Figure S5.*** Size distribution of CJ-Pt/Au BNCs series catalysts prepared by replacement reaction method.


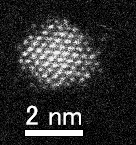


***Figure S6.*** HADDF-STEM images of a CJ-Pt/Au BNC (The red circle in the images indicates the presence of the vacancy resulted from replacement reaction process between Pt mother clusters and Au^3+^ ions. The green dashed square indicates the shape of the NC.).

(a) XPS spectrum of CJ-3 BNCs

(b) Au 4f (c) Pt 4f

***Figure S7.*** Au 4f and Pt 4f XPS core level spectra recorded from CJ-3 BNCs (*R*_PVP_=100).

**References**

[S1] H. J. Zhang, T. Watanabe, M. Okumura, M. Haruta, N. Toshima, Nature Mater., 2012, 11, 49-52.

[S2] H. J. Zhang, L. L, Lu, K, Kawashima, M, Okumura, M, Haruta, N, Toshima, Adv. Mater., 2015, 27(8): 1383-1388.
